# The effect of fear of progression on quality of life among breast cancer patients: the mediating role of social support

**DOI:** 10.1186/s12955-021-01816-7

**Published:** 2021-07-10

**Authors:** Yue Ban, Mengyao Li, Mingli Yu, Hui Wu

**Affiliations:** Department of Social Medicine, School of Public Health, China Medical University, No. 77 Puhe Road, Shenyang North New Area, Shenyang, 110122 Liaoning China

**Keywords:** Breast cancer, Quality of life, Fear of progression, Social support

## Abstract

**Background:**

Women with breast cancer are prone to have mental stress and be stimulated by the fear of progression (FOP), then giving rise to a lower quality of life (QOL). The study aimed to examine the relationships between FOP, social support and QOL, and further explore whether social support mediates the association between FOP and QOL among Chinese patients with breast cancer.

**Methods:**

The cross-sectional study was conducted from October 2019 to May 2020 at Anshan Cancer Hospital in Liaoning, China. 244 female breast cancer patients completed questionnaires including the Functional Assessment of Cancer Therapy for Breast (FACT-B), Multi-Dimensional Scale of Perceived Social Support (MSPSS), and Fear of Progression (FOP). Hierarchical multiple regression analysis was performed to assess the associations between FOP, social support and QOL. Asymptotic and resampling strategies were used to explore the mediating role of social support.

**Results:**

The mean QOL score was 90.6 ± 17.0 among the patients with breast cancer. FOP was negatively correlated with QOL, while social support was positively related to QOL. Social support partly mediated the association between FOP and QOL, and the proportion of the mediating effect accounted for by social support was 25%.

**Conclusions:**

Chinese breast cancer patients expressed low QOL. Social support could mediate the association between FOP and QOL. Medical staffs and cancer caregivers should alleviate patients’ FOP to improve their QOL by facilitating social support.

## Background

Breast cancer is one of the most common and fatal diseases in the female population, which has a great impact on the psychological, emotional, social and family life of breast cancer patients [1]. In China, the incidence of breast cancer is growing at a rate twice as compared to that of global cancer rate of increase [2]. Besides, female breast cancer showed an escalating trend in years of life lost from 1990 to 2017 [3].The increase of morbidity in breast cancer afflicted patients, and aggravation for disease burden put a long-term impact on the psychological health of patients [4]. Simultaneously, psychological distress or emotional changes also have a negative influence on quality of life (QOL), which of the impact is not only confined to the treatment phase but also in the post treatment period [5].

QOL is generally considered to be a multidimensional concept that includes physical, psychological, and social well-being, feelings of health as well as symptoms associated with illness or treatment [6]. It has already been an important indicator which monitors the process of cancer treatment and prognosis or rehabilitation effect in recent years [7]. With the development of medical technology, the 5-year survival rate of breast cancer patients was as high as 68.1–93.2% [8]. Even so, due to the pathological and physiological characteristics of cancer itself, the progression or recurrence of cancer has not been effectively solved and prevented [9]. The recurrence rate of breast cancer patients in China was up to 5–30% [10]. The high recurrence rate may bring psychological stress to patients and impair their QOL [11]. Additionally, Chinese traditional culture stems from interdependent values that emphasize the importance of maintaining prestige and social status [12]. Diagnosis of cancer might potentially affect patients’ expression and talk about their fear of cancer. Many patients did not receive adequate psychological support when confronted with the fear of illness progression or recurrence [13]. Furthermore, negative psychological distress and unmet social demands were significantly associated with poor QOL [14]. Consequently, the effect of internal psychological state and external social factors on QOL in breast cancer patients should be given enough attention.

Fear of progression (or recurrence, hereafter FOP) is defined as the fear of the illness progressing or recurring in the same place or in another part of the body [9]. FOP has become an important psychological burden of breast cancer patients, which seriously affects their QOL [15]. Foreign scholars showed that probably 50% of cancer survivors experience moderate to severe FOP [9]. Moreover, a longitudinal study of QOL for early-stage breast cancer survivors presented that the poorer QOL in women was related to long-term worry about cancer progression [16]. FOP can lead to decrement in physical, mental and social aspects of QOL as well as persist longer after the completion of an active treatment [17]. However, to the best of our knowledge, few studies [15] have examined the relationship between FOP and QOL among female breast cancer patients. Therefore, it is essential to implement relevant research.

Facilitating positive psychosocial outcomes is as crucial as the decreasing negative ones. One of the most effective ways to cope with a traumatic life event is social support [18] which is commonly defined as informational, emotional, and useful assistance provided by one’s social network [19]. It played a key role in promoting positive psychological outcomes among breast cancer survivors [20]. The significance of social support has been justified by You et al. that it was successfully used in adjusting to cancer [21]. Surveys of different cancer populations demonstrated that higher social support was linked to better QOL [22, 23]. It was also found social support could alleviate psychosocial distress, thereby holds the potential to serve as a buffer against FOP as well [24]. Additionally, influenced by Chinese Confucian family harmony, Chinese female breast cancer patients would rather prefer to bear the disease pressure alone because of being afraid of bringing a burden to others [25]. Whereas, some scholars indicated that emotional support from family members, which is naturally expected from a closer family members such as spouse and children, have a positive effect on the mental and physical adaptation to the disease [26, 27]. Internal and external positive coping strategies from society or family support are vital for patients to readjust with their surrounding changes [28]. Leung et al. indicated that social support could help patients improve their mental health and enhance their confidence to better cooperate with treatment [29]. Hence, it is necessary to study the role of social support on QOL of breast cancer patients to help alleviate the fear of disease recurrence and pressure of cancer.

Up to now, although the relationships among FOP, social support and QOL have been reported previously [15], the mediating effect of social support between FOP and QOL for Chinese breast cancer patients have not been clearly examined. Social support might be regarded as an important mediating variable in interpersonal relationship, the role mainly relied on individuals’ ability to express their needs [30]. Celik et al. found that social support might transform patients’ FOP and help them cope with uncertainty, in which way the patients had a better performance in adapting to society and in turn improved their QOL [31]. Walsh assessed the mediating effect of social support by identifying the linkage between distress and QOL for breast cancer patients, the result indicated that social support partially mediated the relation between symptom distress and QOL [32]. The above researches suggested that social support might help cancer patients ease the negative emotions and boost QOL.

Based on the above literature studies, we hypothesized that social support was an important factor in improving QOL. Given that FOP is common among breast cancer patients [16, 17, 33] and may generate adverse consequences, hence, if social support is a mediator mechanism between FOP and QOL, it will provide significant intervention guidance for buffering the adverse effects of FOP on QOL. In view of the empirical studies above and different cultural context and conditions, the purpose of our study was to verify the following three hypotheses among Chinese female breast cancer patients: 1) FOP has a negative effect on QOL; 2) social support has a positive effect on QOL; 3) social support mediates the association between FOP and QOL.

## Methods

### Ethics statement

The procedures of this study were reviewed and approved by the Committee on Human Experimentation of China Medical University and Anshan cancer hospital in Liaoning, China. And the process of study was in accordance with the ethical standards.

Written informed consent for the investigation was obtained from each participant. We protected personal privacy when handling personal data and kept personal records confidential.

### Participants and procedure

A cross-sectional study was conducted from October 2019 to May 2020. All participants were from the Department of Breast Surgery, Anshan Cancer Hospital, Liaoning, China. The inclusion criteria in this study were as follows: (1) Chinese speaking female having age ≥ 18 years; (2) with pathological diagnosis of breast cancer at any stage of disease; (3) had completed all surgical procedures and continued radiotherapy or chemotherapy; (4) had clear consciousness and cognition. Exclusion criteria were as follows: (1) patients suffered from mental problems or cognitive disorders and intellectual impairments prior to cancer diagnosis; (2) unwilling to be enrolled into the study program. Self-administered questionnaires were distributed to the patients by researchers and medical staff, which were being given rigorous training beforehand prior the survey. The process of collecting questionnaires had strict quality control measures to avoid possible bias. Eligible patients would sign an informed consent form and filled out the questionnaire in a private place in the inpatient department within one week after surgery. A total of 266 patients had given consent and were enrolled by research staff to assess for eligibility. Twenty-two patients were excluded due to missing values exceeding 10% (mainly cancer stage and various items of the QOL questionnaire). Finally, 244 breast cancer patients were admitted into the analysis with an effective response rate of 92%.

### Demographic and clinical characteristics

There were six demographic variables and four clinical variables in our study. Age at time of survey was divided into three types: “≤ 45”, “46–55” and “≥ 56” [34]. Residence was divided into two groups: “city” and “rural”. Marital status included two groups: “single/separated/divorced/widow” and “married/cohabitation”. Education level was categorized as “middle school or under”, “senior high school”, “undergraduate or above”. Household per capita monthly income (RMB: Yuan) included “≤ 3000”, “3001–4000”, “4001–5000” and “≥ 5001” [35]. Principal caregiver comprised spouse, adult children and relatives. Cancer stage were divided into “0-I”, “II” and “III + IV”. Others (current recurrence, chemotherapy, radiotherapy) were divided as “yes” and “no” two groups.

### Measures

#### Measurement of QOL

The Chinese simplified version of the FACT-B was used to assess the QOL, which has shown good reliability and validity [36]. It comprised five subscales: physical (seven items), social (seven items), emotional (six items) and functional well-being (seven items) together with the Breast Cancer Subscale (BCS) (nine items). Each item was given on a five-point Likert scale (0 = “not at all” to 4 = “very much”). A total score of QOL was obtained by summing the scores of all five subscales [37]. The total score ranges from 0 to 144 and patients with higher scores suggest better QOL. The Cronbach’s alpha coefficient of total scale was 0.879 in present study.

#### Measurement of the fear of progression

Fear of progression was measured by the 12-item short version of the Fear of Progression Questionnaire (FOP-Q-SF) [38]. And each item was rated on a 5-point Likert scale ranging from 1 “never” to 5 “very often”. Higher scores indicate a higher fear of progression. Wu et al. [39] translated it into the Chinese version and had a high internal consistency coefficient (Cronbach’s alpha = 0.883). In the current study, the Cronbach’s alpha for the total scale was 0.945.

#### Measurement of social support

We chose the Multi-Dimensional Scale of Perceived Social Support (MSPSS), which was measured by using the 12-item version designed from Zimet et al. [40]. The scale comprised three measurements: families and friends support as well as significant others. The gross score of social support was used in current sample. Participants rated each item on a 7-point Likert scale (1 = “very strongly disagree” to 7 = “very strongly agree”). The higher total score indicates better social support. The Chinese version of the scale had been verified adequate reliability and validity among cancer patients [41] and Cronbach’s alpha coefficient was 0.965 in our research.

### Statistical analysis

All the analyses were performed by IBM SPSS Statistics 21.0 (IBM, Asia Analytics Shanghai), with a two-tailed *P* < 0.05 considered to be statistically significant. Before conducting the data analyses, the normal distribution of the variables was tested by P–P-plot analyses and Kolmogorov–Smirnov tests. The QOL and continuous variables fulfilled the postulation of normal distribution in our study (*P* > 0.05). According to demographic and clinical groups, we used t-test and one-way ANOVA analysis to examine group differences of QOL. Pearson’s correlation analysis was applied to analyse the correlations among QOL, FOP and social support. Hierarchical multiple regression analysis was conducted to explore social support as a potential mediating role on the association between FOP and QOL. Regression analysis consisted of four steps. In step 1, the age and potential control variables (which were significant variables in univariate analysis) were entered, FOP was entered in step 2 and social support was entered in step 3, FOP and social support simultaneously were entered in step 4. The variance inflation factor (VIF) values < 10 were considered to be non-collinear [42].

We used asymptotic and resampling strategies to examine whether social support mediated the association between FOP and QOL [43]. Our study was performed by five thousand bootstrap samples. The selection of control variables was based on the statistical significance of univariate analysis. Total scores for FOP, social support and QOL were standardized separately by subtracting the mean value and dividing by the standard deviation to account for the differences in scale scores. The total effect (“c path”), the direct effect (“c’ path”) and the indirect effects (“a*b path”) were presented. The bias-corrected and accelerated 95% confidence interval (BCa 95% CI) for each a * b product was calculated and if a BCa 95% CI excluded 0, indicating a significant mediation.

## Results

### Descriptive statistics

Demographic and clinical characteristics of participants and group differences on QOL were shown in Table 1. The average age of the participants was 54.3 ± 10.5 (mean ± SD) and 121 (50%) of them were above 56 years old. 102 (42%) of patients had senior high school and 115 (47%) of patients were cared by relatives. With regard to clinical variables, only 27 (11%) of the patients had undergone cancer recurrence. Among the six demographic variables, education level and principal caregiver were found to be significantly correlated with QOL, and patients who went senior high school reported higher QOL score than those who went middle school or under and undergraduate or above (*P* < 0.05); and patients cared by different caregivers also showed different levels of QOL (*P* < 0.05). Among the four clinical variables, only whether current recurrence or not was found to be significantly correlated with QOL, and patients who had undergone cancer recurrence reported lower QOL score than those without recurrence (*P* < 0.05).

**Table 1:** Demographic and clinical characteristics and the score of QOL among breast cancer patients

Variables	N (%)	QOL			
		Mean	SD	*F/t*	*P*-value
Age				2.739	0.067
≤ 45	47 (19)	91.7	18.0		
46–55	76 (31)	86.8	16.1		
≥ 56	121 (50)	92.5	16.8		
Residence				− 0.103	0.918
City	192 (79)	90.5	17.1		
Rural	52 (21)	90.8	16.6		
Marital status				1.700	0.090
Married/cohabitation	224 (92)	91.1	16.7		
Single/divorced/Separated/widow	19 (8)	84.4	18.5		
Education level				3.186	0.043
Middle school or under	86 (35)	91.8	17.1		
Senior high school	102 (42)	92.2	16.2		
Undergraduate or above	56 (23)	85.6	17.5		
Income (Yuan per Month)				1.671	0.174
≤ 3000	78 (32)	90.9	19.0		
3001–4000	88 (36)	92.4	16.5		
4001–5000	29 (12)	91.9	14.2		
≥ 5001	49 (20)	85.9	15.5		
Principal caregiver				4.804	0.009
Spouse	53 (22)	90.7	18.4		
Adult children	76 (31)	95.1	15.2		
Relatives	115 (47)	87.8	16.8		
Current recurrence				− 2.580	0.01
Yes	27 (11)	82.7	14.3		
No	217 (89)	91.5	17.0		
Cancer stage				0.074	0.929
0–I	61 (25)	91.3	17.1		
II	109 (45)	90.32	17.0		
III + IV	74 (30)	90.3	17.1		
Chemotherapy				− 0.316	0.752
Yes	159 (65)	90.3	16.9		
No	85 (35)	91.0	17.2		
Radiotherapy				− 1.859	0.064
Yes	98 (40)	88.1	15.9		
No	146 (60)	92.5	17.5		

### Correlation between FOP, social support and QOL

Correlation coefficients between continuous variables were presented in Table 2. The mean QOL score among breast cancer patients was 90.6 ± 17.0. FOP was negatively associated with QOL (*r* = − 0.408, *P* < 0.01), social support was positively correlated with QOL (*r* = 0.472, *P* < 0.01).

**Table 2:** Correlation between FOP, social support and QOL

Variables	Mean	SD	QOL	FOP	Social support
QOL	90.6	17.0	–		
FOP	37.8	8.8	− 0.408**	–	
Social support	60.6	11.8	0.472**	− 0.239**	–

**P < 0.01

### Hierarchical multiple linear regression

The results of the hierarchical multiple regression were shown in Table 3. FOP and social support totally accounted for 29% of the variance in QOL. FOP was negatively associated with QOL (*β* = − 0.391, *P* < 0.01; step 2) and social support was positively associated with QOL (*β* = 0.472, *P* < 0.01; step 3). Moreover, the absolute value of regression coefficient of FOP on QOL diminished from 0.391 to 0.293 (*β* = 0.293, *P* < 0.01) after adding social support in step 4. The results meant that social support probably mediated the relation between FOP and QOL partially.

**Table 3:** Hierarchical multiple regression results of QOL among breast cancer patients

Variables	QOL											
	Step 1			Step 2			Step 3			Step 4		
	*β*	*SD*	*P*-value	*β*	*SD*	*P*-value	*β*	*SD*	*P*-value	*β*	*SD*	*P*-value
Constant	81.898			108.867			39.780			66.336		
Age												
≤ 45	0	–	–	0	–	–	0	–	–	0	–	–
46–55	− 0.170	0.464	0.047	− 0.165	0.464	0.036	− 0.134	0.464	0.074	− 0.136	0.464	0.056
≥ 56	− 0.133	0.501	0.165	− 0.138	0.501	0117	− 0.093	0.501	0.272	− 0.102	0.501	0.200
Education level												
Middle school or under	0	–	–	0	–	–	0	–	–	0	–	–
Senior high school	0.018	0.494	0.803	0.087	0.494	0.185	0.002	0.494	0.976	0.056	0.494	0.343
Undergraduate or above	− 0.115	0.421	0.127	− 0.044	0.421	0.532	− 0.100	0.421	0.132	− 0.049	0.421	0.441
Principal caregiver												
Spouse	0	–	–	0	–	–	0	–	–	0	–	–
Adult children	0.095	0.464	0.261	0.098	0.464	0.210	0.197	0.464	0.009	0.183	0.464	0.011
Relatives	− 0.101	0.500	0.216	− 0.060	0.500	0.430	0.007	0.500	0.927	0.022	0.500	0.752
Current recurrence												
Yes	0.139	0.314	0.028	0.132	0.314	0.023	0.110^*^	0.314	0.047	0.109	0.314	0.037
No	0	–	–	0	–	–	0	–	–	0	–	–
FOP	0	–	–	− 0.391	8.758	0.000	0	–	–	− 0.293	8.758	0.000
Social support	0	–	–	0	–	–	0.472	11.769	0.000	0	–	–
Social support	0	–	–	0	–	–	0	–	–	0.401	11.769	0.000
*F*	3.238		0.003	8.891		0.000	12.594		0.000	15.709		0.000
Adjusted R^2^	0.061			0.206			0.276			0.353		
ΔR^2^	0.088			0.145			0.212			0.144		

### Asymptotic and resampling strategies in the mediating role of social support and its path analysis

**Fig. 1:**
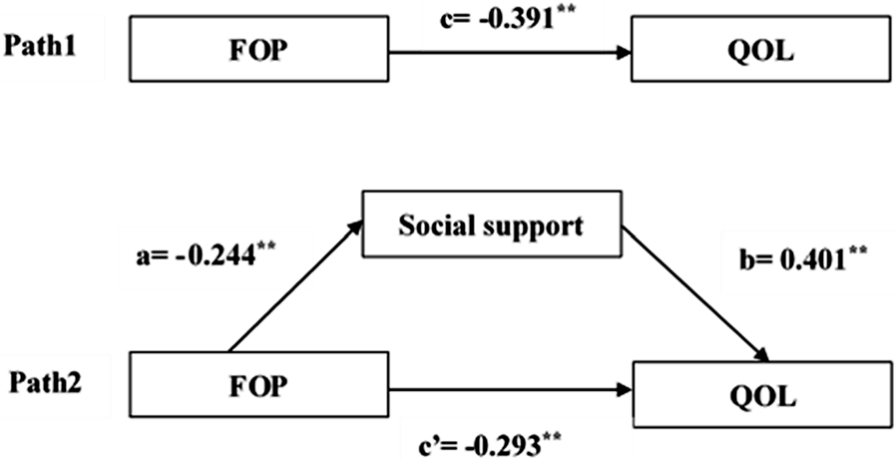
Model of the mediating role of Social support between FOP and QOL. *Note*: **P < 0.01

The results of the mediation analysis were presented in Table 4. The total effect of FOP on QOL (“c path”) was initially evaluated. FOP was negative association with QOL (c = − 0.391, *P* < 0.01). Then, the indirect effect of FOP on QOL via social support was observed (path a * b, a = − 0.244, b = 0.401, a * b (BCa 95% CI) = − 0.098 (− 0.161, − 0.038)). The confidence interval for indirect effect did not contain zero, which suggested that social support played a mediating role between FOP and QOL. Furthermore, when social support was entered to the model as a mediator, the direct effect of FOP on QOL (path c′) was still significant (c’ = − 0.293, *P* < 0.01). Hence, social support had a partially mediating effect in the relationship between FOP and QOL for patients in this study. To understand the effect size of the mediating pathway, we calculated the proportion of the total effect of the FOP on QOL that was mediated by social support with the formula (a*b)/c. The proportion of mediation of social support was 25%. The mediating model and path coefficients were shown in Fig. 1.

**Table 4:** The results of the mediation analysis

Mediation path	Coefficient	*P-*value	BCa 95% CI
c	− 0.391	< 0.01	(− 0.506, − 0.275)
a	− 0.244	< 0.01	(− 0.369, − 0.119)
b	0.401	< 0.01	(0.293, 0.508)
a * b	− 0.098	–	(− 0.161, − 0.038)
c′	− 0.293	< 0.01	(− 0.401, − 0.185)

BCa 95% CI: the bias-corrected and accelerated 95% confidence interval; age, education level, principal caregiver and current recurrence were covariates

## Discussion

Our findings showed that the mean score of QOL value was 90.6 ± 17.0 in the present study, which was lower compared with the level of QOL (96.4 ± 17.7) reported in Eastern China among female patients with breast cancer [44]. Moreover, the mean score of QOL value was much lower than that of patients with breast cancer in other developed countries. Milbury et al. reported that the mean score of QOL was 104.12 ± 14.77 in USA during the year 2017 [45], Matthies et al. reported a mean QOL score of 102.66 ± 22 in Germany during the year 2019 [46]. However, the mean QOL score observed in our investigation was closer to the Asian situation-the mean score of QOL in Korea was 91.26 ± 20.08 as reported by Park et al. in 2019 [47]. QOL in the current study was at a lower level in its individual capacity possibly because of three reasons. Firstly, although there is continuous improvement in diagnosis and treatment technology and it is more convenient to obtain quality care in China, the psychological fear brought by the cancer itself and unmet inner needs of patients have not been received adequate attention by nursing staff and family members [48]. Secondly, Chinese cultural philosophies shape individual perceptions of disease, which are completely different from western cultural foundations. Influenced by traditional introvert culture, Chinese people are unwilling to share their feelings and thoughts with others, especially regarding disease-related issues [49]. As a result, some emotional pressure cannot be released contributing to psychological disorders of Chinese female breast cancer patients. Particularly, surgery and relevant treatments (changes in sexual function and self-image) affect an individual’s physiology, psychology and social-relations [50], thus promoting a lower QOL. Finally, subsistent medical institutions cannot offer targeted professional consultation to cancer patients in China, especially patients with recurrence, which is one of the most frustrating and difficult phase for cancer patients. Karam proposed that most recurrences are symptomatic (e.g. chest pain, cough, dyspnea, and so on) and occur during the interval between scheduled visits [51]. Relapsed patients had a higher sense of fear of progression at reexamination time, together with disturbing thoughts, anxiety and poor QOL [52]. By contrast, psychological counseling and supportive care are widely popularized in western countries [53]. The above-mentioned conditions collectively intensified the negative impact on patients, which led to the poorer QOL of breast cancer patients.

Furthermore, the associations between FOP, social support and QOL of breast cancer patients were explored in our study. We found that FOP was negatively associated with QOL. Gotze et al. demonstrated that the FOP was associated with reduced emotional and social domains on QOL [54]. Socio-demographic, psychosocial and clinical characteristics, such as age, living with a spouse, social support and poorer health conditions were important determinants of FOP [55]. Gallenkamp et al. indicated that female survivors who were in five to seven years post-diagnosis, being socially isolated and those having less education or recurrence were at a greater risk to experience moderate or high levels of FOP [24]. These factors had an impact on the QOL, reducing life expectancy as well. What is more, our results indicated that social support positively correlated with QOL among patients with breast cancer. Cancer patients revealed higher QOL and lower depression when they received more social support [56]. Besides, in close relationships, women’s adaptation to breast cancer and family expression patterns significantly affect patients’ ability to cope with the disease [57]. Lim et al. quoted that effective communication and support contributed to a higher QOL [58]. As a consequence, medical staff and family caregivers must be aware of the importance of providing social support to breast cancer patients.

As expected, the result showed that social support acted as a mediator in the relation between FOP and QOL among Chinese breast cancer patients. A higher level of FOP might be alleviated by the higher level of social support and further led to the higher level of QOL. Social support (as a positive factor) weakened the negative impact of FOP (as a risk factor) on QOL. In brief, social support played a significant role in patients’ health outcomes including health-related quality of life [29]. It meant we could improve the QOL of breast cancer patients via promoting the level of social support and reducing the sense of fear of disease. For this purpose, designing intervention programs via enhancing social support is an effective way to eventually achieve the goal of prolonging life expectancy and improving QOL. A supportive-expressive intervention program developed by the Stanford university laboratory proposed a kind of intervention model including social support which is mainly focused on encouraging emotional expression, arranging life priorities, dealing with a fear of death, cultivating relationships with families and friends, and increasing the adaptability to cope with a traumatic event and eventually improve the QOL [59]. Kissane et al. using this intervention model on Australian patients with metastatic breast cancer, evidenced that the effectiveness of this model in improving QOL and suppress depressive emotions was significant [60]. Therefore, future studies should be focused on the impact of interventions related to social support on Chinese breast cancer patients.

There were several limitations of our study. Firstly, a cross-sectional design was applied to the present study, so these findings could not be used to construct a formal causality or to identify the direction of causality between psycho-social resources and QOL. They are needed to be validated via longitudinal researches. Secondly, individuals with other types of diseases or variable comorbidities might not be surveyed. Additionally, we only recruited breast cancer patients from a single hospital in Liaoning Province, north of China, which might limit its applicable to other regions due to the cultural differences between North and South China. Thirdly, psychological variables were mainly evaluated using self-report instruments, which might be subject to recall and response bias. Our study tried to minimize the bias by using the QOL, FOP, and MSPSS that have been well validated for application among subjects in China. Finally, our study focused only on the association between FOP, social support and QOL. Further investigation needs to be taken into consideration to explore other social psychology and emotional predictors for the level of QOL in breast cancer patients, such as society, family environment factors and so on.

## Conclusions

In summary, our findings suggested that the QOL of breast cancer patients was generally at a lower level in Liaoning Province, China. Social support partially mediated the relationship between FOP and QOL in breast cancer patients, which was the first attempt to perform the relationship between psycho-social mediating resources and QOL among Chinese breast cancer to our limited knowledge. Present study highly recommended that positive social support would be beneficial to improve the QOL of breast cancer patients. Meanwhile, providing targeted support for the breast cancer patients, such as positive interventions of expressive support, might be helpful to improve their QOL as well as relieve their fear of disease in the oncology field.

## Data Availability

The datasets used and/or analyzed during the current study are available from the corresponding author on reasonable request.

## Abbreviations

QOL: Quality of life
FOP: Fear of progression
MSPSS: Multi-Dimensional Scale of Perceived Social Support
FACT-B: Functional Assessment of Cancer Therapy for Breast
